# Leech Me Alone! Atraumatic Hemarthrosis after Hirudotherapy

**DOI:** 10.7759/cureus.6915

**Published:** 2020-02-07

**Authors:** Janine Curcio, Christopher M Lloyd

**Affiliations:** 1 Emergency Medicine, OhioHealth Doctors Hospital, Columbus, USA

**Keywords:** hemarthrosis, hirudotherapy, leech

## Abstract

A 58-year-old male presented to the emergency department with a chief complaint of knee pain and swelling after performing hirudotherapy (leech therapy) approximately one week prior. Knee arthrocentesis demonstrated significant hemarthrosis. Hirudotherapy is being used for a broad array of reasons including treatment of osteoarthritis, to plastic and reconstructive surgery. Case reports and journal articles often discuss cutaneous reactions, bleeding, and infection as common adverse events. Intra-articular bleeding is not commonly mentioned. With hirudotherapy being utilized more as alternative therapy for osteoarthritis and joint pain, physicians should be aware of hemarthrosis as a possible adverse reaction.

## Introduction

Hirudotherapy, the use of leeches in medical treatment, began as early as 200-130 BC [1]. Initially it was used for bloodletting and ‘balancing the biological humors’ [2]. Then in the 19th century, leeches were used for treatment of hematomas, hemorrhoids, headaches, and abscesses. With the advent of modern medicine, hirudotherapy initially had a decline but now over the last few decades has had a resurgence. Today, it is used to stimulate blood circulation after plastic surgery, heal wounds, and relieve the pain associated with osteoarthritis. A single bite of the leech, Hirudo medicinalis, releases 100 different bioactive materials. A small example of these materials are anti-coagulants like hirudin and factor Xa inhibitors, vasodilators like acetylcholine and histamine like substance, and hyaluronidase that increases intestinal viscosity and antibiotic action [2]. It is suggested that hirudotherapy’s mechanism of action includes a wide range of secretions that ultimately eliminate micro-circulation disorders, restore vascular permeability of tissues, eliminate hypoxia, and improve the bioenergetic status of the organism [2-4]. In regards to treatment of osteoarthritis it is thought that the hyaluronidase allows the anti-coagulants, vasodilators, and anesthetics to reach the deep tissues and the joint space providing anti-inflammatory effects and pain relief. As the use of leeches in modern medicine is increasing the most common adverse reactions from leech therapy include infection, cutaneous bleeding, and allergic reactions [2]. A meta-analysis summarizing the use of Hirudo medicinalis, the medicinal leech, for osteoarthritis reported adverse reactions being more cutaneous in nature with localized itching, cellulitis, and mild bleeding. It did not note any intraarticular side effects from this therapy [5]. After an extensive publication search there was only one case report of atraumatic hemarthrosis after hirudotherapy, but the patient was on coumadin as well [6]. We present what we believe to be the first reported case of atraumatic hemarthrosis of a patient not on antiplatelet or anticoagulation after H. medicinalis therapy.

## Case presentation

A 58-year-old male with past medical history of diabetes mellitus presents to the emergency department with a chief complaint of knee pain and swelling for one week. He stated approximately six weeks ago he fell and injured his left knee. Due to persistent pain, the patient underwent hirudotherapy approximately one week ago. Since then he has had worsening joint swelling and pain. He reports difficulty with bending due to swelling. He denies difficulty ambulating, secondary trauma, surgeries and blood thinner use. He denies fever, chills, rashes and erythema.

Physical exam revealed edema and obvious effusion of the left knee. There was no erythema, rash, or warmth appreciated. The patient could range his knee to 90 degrees without pain, but difficulty ranging further secondary to swelling. There was tenderness to palpation of the knee joint. Right knee was unremarkable. Lower extremity had soft compartments, strong pulses, and brisk capillary refill. Muscle strength was 5/5 and sensation intact to light touch bilaterally.

**Figure 1:**
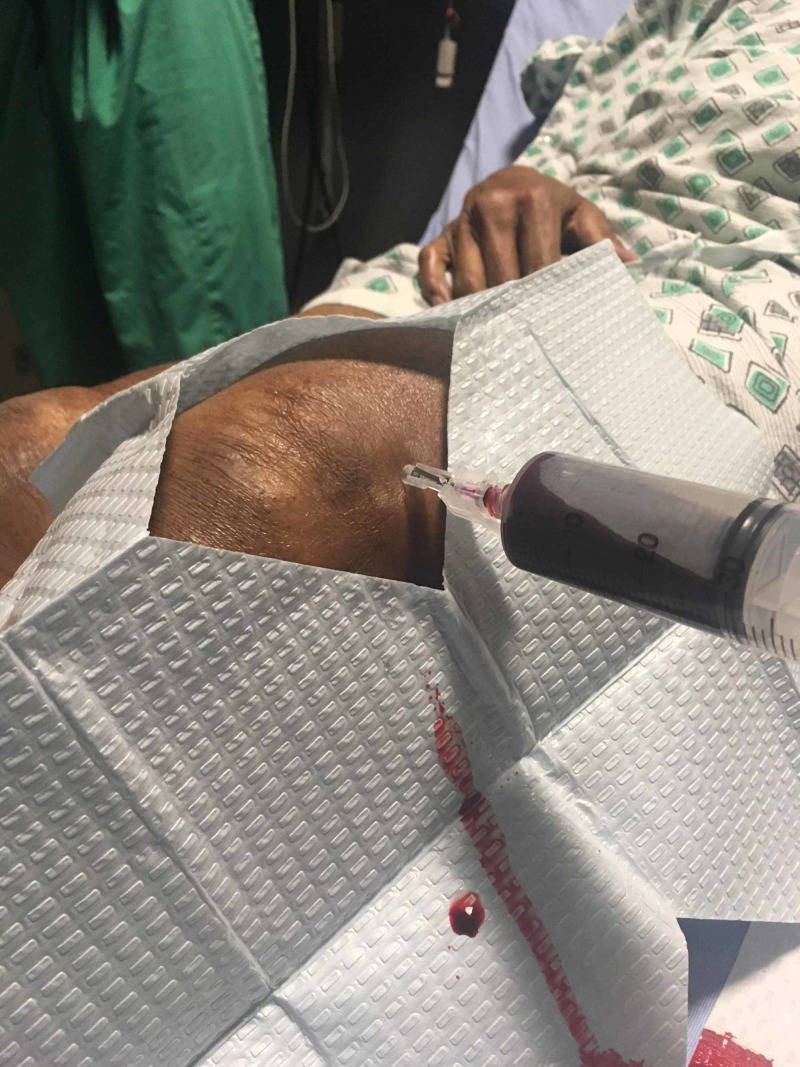
Arthrocentesis

A left knee X-ray was obtained and demonstrated large suprapatellar joint effusion, extensor mechanism enthesopathy and ossific densities in the suprapatellar fossa possible related to osteochondromatosis. Lab work was obtained including complete blood count (CBC) and erythrocyte sedimentation rate (ESR) which demonstrated no leukocytosis and mildly elevated ESR at 22. Arthrocentesis was performed and approximately 90 ml of blood was aspirated from the joint (Figure 1). Synovial fluid analysis is presented in Table 1. Synovial fluid cultures were negative. The patient was discharged home with ace wrap and 48-hour recheck.

**Table 1:** Synovial Fluid Analysis WBC: White blood cell; RBC: Red blood cell; SNF: Synovial fluid.

	Reference Range and Units	Results
Volume	mL	60
Color, SNF	Straw	Red
Appearance, SNF	Clear	Bloody
Fibrin Clot, SNF	Absent	Absent
Protein, SNF	0.0-3.0 g/dL	6.2
RBCs, SNF	0-0/mcL	>1,000,000
WBC/Nucleated Cells, SNF	0-200/mcl	1,879
Neutrophils, SNF	0-25%	67
Lymphocytes, SNF	0-0%	28
Monocytes, SNF	0-0%	2
Eosinophils, SNF	0-0%	3
Urates, SNF	Negative	Negative
Calcium Pyrophosphate, SNF	Negative	Negative

## Discussion

Leech therapy was first approved by the Food and Drug Administration for graft tissue healing in 2004, and since then, the use of leech therapy is expanding into alternative methods of treatments of joint pain including knee pain from osteoarthritis [1,7-9]. Leeches have over 100 bioactive substances that work synergistically. It is thought the penetration of these substances into the joint space provides anti-inflammatory properties and pain relief. In 2018, a meta-analysis of controlled trials was performed reviewing the safety and efficacy of hirudotherapy for osteoarthritis. They reported the most common adverse reaction was local itching and skin redness with three cases of cellulitis. There were also two cases with mild reduction in systolic blood pressure, resulting in lightheadedness and dizziness. Mild bleeding was reported without any cases of prolonged bleeding [5]. There was no mention of hemarthrosis or systemic bleeding.

There are case reports discussing adverse bleeding after hirudotherapy, but often the patient is already on a drug that disrupts the coagulation profile. For example, a 50-year-old male presented for hematemesis that began two hours after leech therapy. It was his second treatment with leeches in four days for knee pain. The patient was on aspirin therapy for coronary artery disease, and his endoscopy revealed erosive gastritis with evidence of bleeding. It is known that aspirin can cause erosive gastritis as well as gastrointestinal bleeding, but it is hypothesized that this patient’s leech therapy was a precipitating factor for the bleeding as hematemesis started a few hours after therapy [10]. There is also an example of bilateral, non-traumatic hemarthrosis from hirudotherapy. A 64-year-old female presented very similarly to our patient complaining of worsening knee pain and swelling. She was using leeches in treatment of chronic pain. She presented because of the pain and swelling but also because there was unstoppable localized bleeding. The patient was found to have bilateral hemarthrosis, and it was believed due to her concomitant coumadin use [6]. Although it has been determined that leeching did not alter the systemic coagulation profile based on a 1994 study, it has never been studied if anti-coagulants or anti-platelet medications could have a compounding effect with leeching [10-12]. Also, in 2008, a man was found to have significant coagulation profile disturbances after suffering 130 leech bites. He presented to an emergency department with excessive bleeding and his labwork demonstrated a hemoglobin of 9.2 g/dL (reference range (RR) 13.6-17.2 g/dL), a prolonged prothrombin time (PT), partial thromboplastin time (PTT) and an elevated international normalized ration (INR) of 12.46 (RR 0.85-1.2). The patient underwent transfusion of six units of erythrocyte suspension and eight units of fresh frozen plasma [11].

## Conclusions

As practitioners turn to leech therapy as an alternative therapy for the treatment of pain, ED providers need to be aware of the possible adverse events associated with its use. The leech’s bite can cause several complications and treatment is not as benign as one would originally think. As our case demonstrates, one needs to be aware of the multiple bleeding risks associated with hirudotherapy.
